# Association of mental health status with perceived barriers to healthy diet among Bangladeshi adults: a quantile regression-based approach

**DOI:** 10.3389/fpubh.2025.1487107

**Published:** 2025-02-19

**Authors:** A. B. M. Nahid Hasan, Satyajit Kundu, Ishrat Jahan, Tapu Basak, Mahamudul Hasan, Azaz Bin Sharif

**Affiliations:** ^1^Department of Public Health, North South University, Dhaka, Bangladesh; ^2^Department of Public Health Nutrition, Primeasia University, Dhaka, Bangladesh; ^3^Public Health, School of Medicine and Dentistry, Griffith University, Gold Coast Campus, Southport, QLD, Australia; ^4^School of Public Health, University of Queensland, Herston, QLD, Australia; ^5^World Health Organization (Bangladesh), Dhaka, Bangladesh

**Keywords:** healthy diet, perceived barriers, mental health, Bangladesh, quantile regression

## Abstract

**Introduction:**

Maintaining a healthy diet is essential for both physical and mental well-being. This study investigated the association of mental health status with perceived barriers to maintaining healthy diets among Bangladeshi adults.

**Method:**

This cross-sectional study was conducted between January to June 2023 in Bangladesh. A total of 400 adults aged between 18 and 60 years who reside in Dhaka, Chattogram, and Gazipur cities were recruited using a multistage sampling technique. A questionnaire consisting of 12 questions adapted from previous literature was used to assess barriers to healthy diets. Mental health status was measured using the validated DASS-21 scale. A quantile regression-based approach was used to ascertain the association between mental health status and barriers to healthy diets.

**Results:**

The five most frequently reported barriers to a healthy diet were the use of junk food as a reward or treat (56.25%), difficulty in controlling eating habits when with friends (56%), the cost of healthy food (44.5%), difficulty in taking healthy food at work (46.5%), and difficult to stay motivated to eat healthy food (25%). The study found that gender, marital status, living arrangement, working hours, and family monthly income were significantly associated with perceived barriers to healthy diets. Mental health status was observed to be associated with barriers to healthy diet scores. Depression (*β* =0.34, 95% CI: 0.17 to 0.51) and anxiety (β =0.14, 95% CI: 0.01 to 0.28) were significantly associated with perceived barrier scores at the 50th quantile. Stress was also significantly associated with perceived barrier scores at the 10th (*β* =0.18, 95% CI: 0.09 to 0.27) and the 25th quantiles (β =0.12, 95% CI: 0.03 to 0.21).

**Conclusion:**

In light of the findings, it is imperative to prioritize the advocacy of policies that integrate mental health services and stress management strategies into public health initiatives.

## 1 Introduction

A healthy diet is defined as a balance of different foods and nutrients for good health and well-being (1). The benefits of maintaining a healthy diet include improved energy levels, better weight management, and reduced risk of illness and diseases (2). Previous study has investigated the relationship between diets and health and observed that a healthy diet is associated with improved health outcomes (3). Li et al. conducted a systematic review of observational studies and revealed that greater adherence to a healthy diet is associated with a lower risk of vulnerable co-morbidities (4).

The ‘State of Food Security and Nutrition in the World 2022’ report estimates that 276 Bangladeshi Taka-BDT. Per day is needed for a person in Bangladesh to afford a nutritious and balanced diet (5, 6). Unfortunately, approximately 57% of the population is unable to bear these expenses (6). Consequently, people tend to choose cheaper, unhealthy food options, which have many negative health impacts (7). Inability to meet the cost can lead to undernutrition; Increased poverty and higher food prices lead to a higher likelihood of food insecurity, thus perpetuating malnutrition (8). According to a recent study conducted in Bangladesh, approximately 20.9% of the population is underweight, 16.4% are overweight, and 3.5% are obese (8).

Despite the many health benefits of maintaining a healthy diet, individuals face barriers to adhering to healthy diets. These barriers may include a lack of knowledge about healthy eating, financial constraints, and time constraints (9, 10). A systematic review conducted in Iran focused primarily on perceived barriers to a healthy diet, revealing that the most frequently reported barriers include lack of time, inconvenience in preparing healthy meals, lower cost of less nutritious fast food, limited availability, higher cost of healthier foods, taste preferences, and lack of nutritional knowledge (11). Hasan et al. found that financial constraints were the most significant barrier to maintaining a healthy diet among adults; with additional factors including knowledge gaps, cultural influences, and societal norms influencing dietary choices and practices (12). Furthermore, prices rise when demand for food exceeds supply, and availability suffers, potentially leading to food insecurity (13). Existing research shows that poverty poses a significant barrier to accessing healthy food options in Dhaka, Bangladesh as many families struggle to afford nutritious meals amidst the recent economic crisis (14). Additionally, this investigation unveiled that the lack of investment in suburban and urban agricultural developments resulted in limited availability of fresh fruits and vegetables, particularly in low-income regions.

The prevalence of mental health problems in Bangladesh varies, with estimates ranging from 6.5 to 31.0% among adults (15). According to a 2020 household mental health survey in Bangladesh, 6.7% of adults have major depressive disorder (MDD), which is higher than the Global Burden of Disease (GBD) estimate (16). Mental health problems may act as a barrier to maintaining a healthy diet. Evidence suggests the interaction between depressive symptoms and a lower likelihood of eating a healthy diet (17). Mental health issues such as depression, anxiety, and stress can lead to unhealthy eating habits, such as overeating and/or skipping meals (18). People with mental health problems may also have a decreased interest in food and a decreased ability to prepare or purchase healthy meals (19). A study found that individuals with depression are more likely to have poor dietary habits, including a lower intake of fruits and vegetables and a higher intake of unhealthy foods (20). Malnutrition and unhealthy dietary habits have also been interrelated to poor mental health, largely due to the central nervous system’s need for key nutrients to maintain optimal function (21).

The extent of unhealthy dietary practices and mental health issues is evident globally, and previous research has demonstrated an interrelationship between these factors (22, 23). This study fills a gap in the existing literature by focusing on urban adults’ barriers to healthy diets, a topic not extensively explored in prior research. While earlier studies have looked into dietary behaviors, they overlooked the specific challenges faced by urban populations, including fast-paced lifestyles, high living costs, and limited access to fresh produce. Additionally, one of the studies on this subject was conducted many years ago, making it outdated. By addressing how mental health conditions influence the perception of dietary barriers and integrating relevant socioeconomic factors, this study offers new perspectives that could inform public health strategies tailored to urban settings (12, 24). Therefore, we hypothesized that there might be a significant association between mental health and barriers to healthy diets among Bangladeshi adults and the objective of this study was to assess the association between mental health status and barriers to healthy diets after adjusting for other socioeconomic variables.

## 2 Materials and methods

### 2.1 Study design and participant’s recruitments

This cross-sectional study was conducted in three city corporations (Dhaka, Chattogram, and Gazipur) in Bangladesh from January to June 2023. Participants of both sexes, between the ages of 18 and 60 years, living in selected areas of Dhaka, Chattogram, and Gazipur city corporations, were included in this research. However, Participants who were injured, in a rehabilitation stage, or unwilling to participate were excluded from the study.

The sample size was calculated using the formula of Cochran’s (n = ((z^2^ × p (1-p))/e^2^)). With a 5% margin of error (e), considering the mostly prevalent perceived barriers to a healthy diet (*p* = 66%) as reported in a previous study (25), and the standard normal variate of 1.96 (z), the required sample size was 358. However, the study team reached a large sample of 565. A total of 78 participants declined to participate in the study due to time constraints, workloads, or other personal reasons. Of the remaining participants, 487 completed the interview, yielding a response rate of 86.20%. Additionally, 20 participants were excluded as they were injured or in rehabilitation. During data cleaning, 67 more cases were excluded due to incomplete interviews, missing values and extreme outliers. Finally, a total of 400 respondents were included in the final analysis.

### 2.2 Sampling and data collection

**Figure 1:**
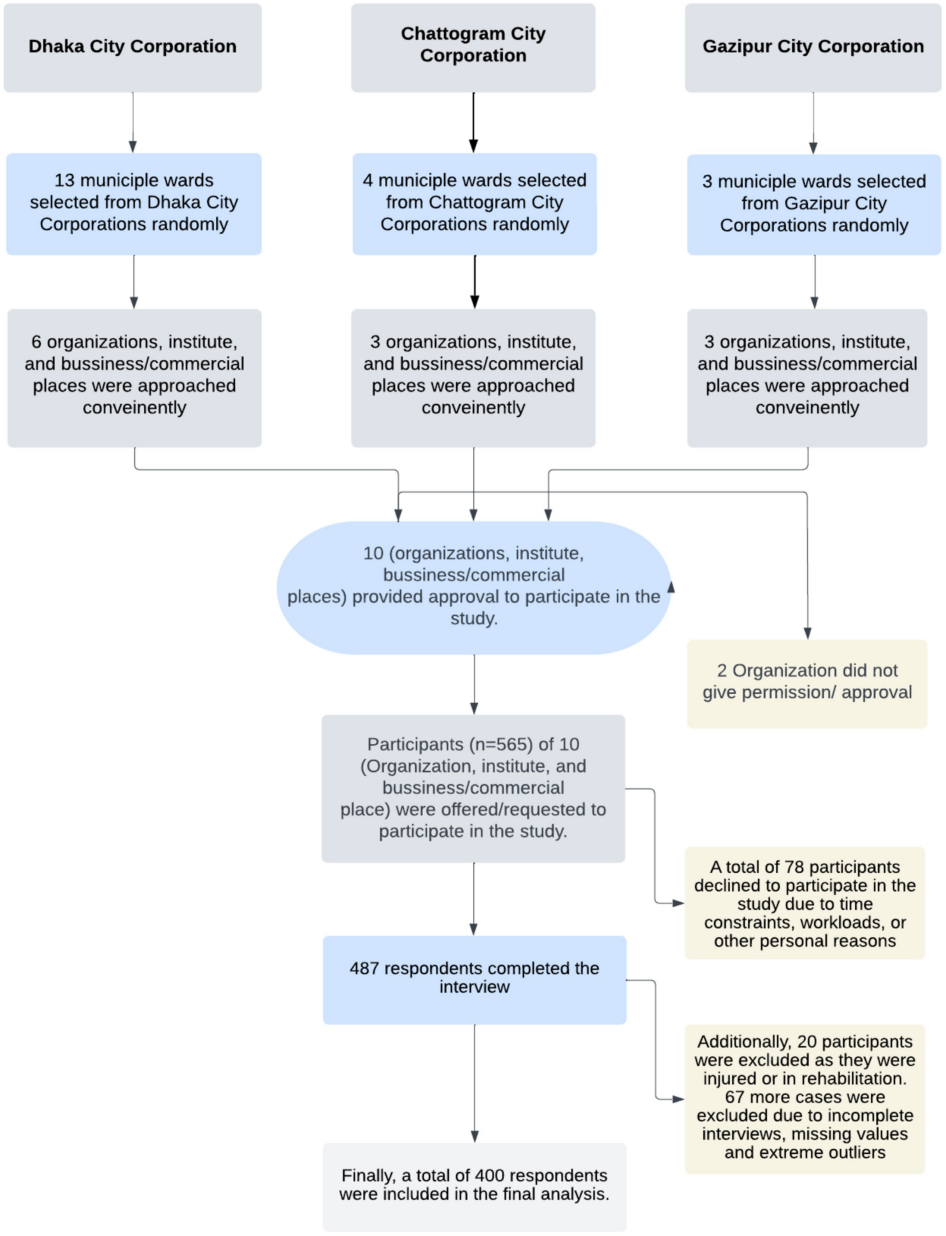
Flow chart of sampling and data collection.

A multistage sampling technique was used to determine the study participants. The details of the sampling procedure have been presented in Figure 1.

In the first stage, 20 wards (sub-division of City Corporation) from three city corporations were randomly selected. At the final stage, study participants were conveniently selected for data collection from each municipal ward. Assessed cities were also represented with map (Supplementary Figure S1).

Five trained surveyor’s/data collectors were used in the data collection process who, in turn, placed in the central business district, shopping malls, and in the educational institutions to capture diverse study population. To recruit data collectors, a circular was issued among recent graduates of the Public Health Department, North South University. Applicants were shortlisted based on qualifications and interviewed. Successful candidates with strong communication skills, research ethics understanding, and fieldwork potential were selected. The principal investigator and a senior research team member provided a three-day training covering study objectives, ethical protocols, consent procedures, questionnaire content, interview techniques, handling sensitive topics, and field protocols for effective and ethical data collection. Participants were approached and a brief description of the study was conveyed. Once participants provided consent to participate in the study, data was collected through face-to-face interviews using a semi-structured and pretested questionnaire. We first formed all of the questionnaires in English, including the questions about barriers to healthy diet. Then, a professional Bengali translator translated them into Bengali. The participants had the choice of using Bengali or English questionnaire. The questionnaire includes socio-demographics, perceived barriers to healthy diet, and mental health-related questions.

### 2.3 Participants

People aged 18 to 60 years living in the Dhaka, Chattogram, and Gazipur city corporations were invited to participate after we approached as much as possible amount of people living in this area. The 18–60 age group is crucial for studying perceived barriers to a healthy diet and their association with mental health. This age range represents a stage in life where individuals typically experience multiple responsibilities, including career development, family obligations, and societal expectations.

### 2.4 Ethical standards disclosure

This study was conducted according to the guidelines laid down in the Declaration of Helsinki and all procedures involving research study participants were approved by the North South University Ethics Review Committee (REF: 2022/OR-NSU/IRB/1003). Written informed consent was obtained from all subjects/patients. Willing respondents participated voluntarily where no financial incentives or gifts were provided to this research due to funding constraints.

### 2.5 Measures

#### 2.5.1 Perceived barriers to healthy diet

Barriers to healthy diet measuring questionnaire was obtained from a previously published study and few of the questions were modified to use in this context (26, 27). Participants were asked 12 questions to assess the perceived barriers to healthy diet using a 5-point Likert scale ranging from “not a problem” to a “significant problem” was used to measure the barriers score. We classified the responses into two groups as newly defined binary variables: those in agreement (answers 4 and 5 options in the Likert scale) and those in disagreement (answers 1, 2, and 3). The response of the barriers scale was also accumulated to the overall score. The reliability value (Cronbach’s alpha) of the perceived barrier to a healthy diet questionnaire was 0.73.

#### 2.5.2 Depression, anxiety, and stress (DASS-21)

The DASS-21 scale is a valid and reliable scale for measuring psychological health. The scale’s reliability coefficient (Cronbach’s alpha) for Depression, Anxiety, and Stress was 0.85. The Cronbach’s alpha values for the subscales are as follows: Anxiety (*α* = 0.7477), Depression (α = 0.7201), and Stress (α = 0.6513). It is a condensed version of the 42-item DASS scale, which consists of the depression, anxiety, and stress subscales (28). There are seven items in each of the three DASS-21 sub-scales. This well-known and widely used DASS-21 scale has been translated and validated in Bengali (29). On a four-point Likert scale, which ranged from 0 (never) to 3 (almost always), respondents were questioned about their level of mental distress over the previous four weeks. Individual depression, anxiety, and stress scores were calculated by summing the scores for their respective 7 items. The final score for each of the 3 dimensions was then multiplied by two to obtain a score between 0 and 42 (30). Individual scores for each of these 3 subscales were then categorized into five severity categories as: normal, mild, moderate, severe, and extremely severe. For depression, scores ranging from 0 to 9 are considered normal, 10 to 13 as mild, 14 to 20 as moderate, 21 to 27 as severe, and 28 or higher as extremely severe. Regarding anxiety, scores between 0 and 7 are categorized as normal, 8 and 9 as mild, 10 to 14 as moderate, 15 to 19 as severe, and 20 or higher as extremely severe. For stress, scores falling between 0 to 14 are considered normal, 15 to 18 as mild, 19 to 25 as moderate, 26 to 33 as severe, and 34 or higher as extremely severe (31).

#### 2.5.3 Socio-demographic status assessments

Participants also filled out questions to attain their socio-demographic data about their age, gender, height, weight, marital status, type of family, education, field of study, occupation, gross monthly household income, and daily working hours. Individuals were subclass into four different groups based on their age. Participants’ self-reported height and weight were used to determine their body mass index (BMI). Then, put into three groups based on the World Health Organization’s-WHO’s major cut-off points: normal range (18.50–24.99 kg/m^2^), underweight (<18.50 kg/m^2^), and overweight and obese (≥25.00 kg/m^2^) (32). Later, the overweight and the obese were combined into one group. Monthly gross household income was used to represent socioeconomic status and put into three groups: <30,000 Bangladeshi currency (BDT), 30,000–60,000 BDT, and > 60,000 BDT. A draft version of the questionnaire in a small sample from Dhaka City has been piloted to evaluate feasibility and acceptability.

### 2.6 Statistical analysis

The STATA (V16 Stata Corp LP, TX, United States) software was used for the analyses. Outliers, identified as extreme values, and missing data were removed to ensure the accuracy of our analysis. Outliers were identified using the interquartile range (IQR) method, where values beyond 1.5 times the IQR from the first or third quartile were considered extreme and removed to minimize skewness in the dataset. For missing data, we used list wise deletion, removing cases with incomplete responses to maintain data integrity and ensure consistent sample size across analyses. Frequencies and percentages were used to narrate the baseline characteristics of the respondents. The Shapiro–Wilk test and a histogram checked the normality of outcome variables. Wilcoxon rank sum test and Kruskal-Wallis test were applied to assess the bivariate analysis as we found our outcome measurements as non-normally distributed. In the multivariate modeling, we adjusted all of the explanatory variables irrespective of their significance in the bi-variate modeling. Quantile regression was used due to the non-normality of our data, offering robust estimates even with violations of normality. We ensured the key assumptions were met, including the absence of multicollinearity, linearity, homoscedasticity, and the correct specification of the model. These diagnostics were conducted following guidelines and practices used in similar studies (33–35).

In the regression modeling, we adjusted for study location, gender, age, current marital status, family type, educational level, field of study, occupation, working hours in a day, family monthly income, BMI, Depression score, Anxiety score, and stress score. Quantile regression analyses were used to figure out how each covariate affected the perceived healthy diet barrier scores on average. A linear regression analysis was also accompanied for comparison purposes. Five quantiles, namely, the 10th, 25th, 50th, 75th, and 90th were used. The hypothesis tests were two-sided, and the *p*-values less than 0.05 were considered significant.

## 3 Results

### 3.1 Socio-demographic characteristics

Table 1 lists the sociodemographic details of the respondents who were chosen from the city corporations of Gazipur (20.5%), Chattogram (29.0%), and Dhaka (50.5%), respectively. There were statistically significant differences (*p* = 0.012) in perceived barriers to a healthy diet between the study locations of Dhaka (23.95 ± 7.90), Chattogram (26.82 ± 9.08), and Gazipur (25.67 ± 7.29). The participants’ mean age was 31.10 ± 10.11, and 66.75% were between 18 and 30 years. The majority of participants were men (68.50%). Around 68.0% of the study population comprised nuclear families, and half of the sample was married.

**Table 1:** Socio-demographic characteristics of the respondents (*N* = 400).

		Perceived barriers to healthy diet score		
Variables	Total; *n* (%)	Mean	SD (±)	*p* value
Study location				
Dhaka city	202 (50.50)	23.95	7.90	0.012*
Chattogram city	116 (29.00)	26.82	9.08	
Gazipur city	82 (20.50)	25.67	7.29	
Gender				
Male	274 (68.50)	25.90	7.65	0.052
Female	126 (31.50)	25.42	10.07	
Age; mean ± SD		31.10	10.11	
18–30 years	267 (66.75)	26.07	8.78	0.610
31–40 years	61 (15.25)	25.11	7.51	
41–50 years	45 (11.25)	24.71	8.57	
51–60 years	27 (06.75)	25.81	7.43	
Current marital status				
Married	194 (51.50)	25.95	7.73	0.180
Single	206 (48.50)	25.54	9.22	
Family type				
Nuclear	272 (68.00)	25.87	8.31	0.002*
Joint family	96 (24.00)	24.00	8.04	
Life apart home	32 (8.00)	30.03	9.71	
Education level				
Higher secondary and below	205 (51.25)	25.06	7.87	0.144
Graduation and above	195 (48.75)	26.48	9.04	
Field of Study				
Biological science	120 (30.00)	26.20	8.59	0.442
Other than biological science	280 (70.00)	25.56	8.44	
Occupation				
Service	137 (34.25)	26.82	9.34	0.097
Business	61 (15.25)	23.88	7.84	
Others	202 (50.50)	25.59	7.96	
Working hours in a day				
6 Hour	19 (4.75)	29.63	12.41	0.341
8 Hour	116 (29.00)	25.90	7.40	
10 Hour and more	99 (24.75)	26.13	9.89	
Not fixed	166 (41.50)	24.98	7.63	
Family monthly income (in BDT)				
<30,000	184 (46.00)	26.11	8.60	0.157
30,000–60,000	147 (36.75)	26.08	8.50	
> 60,000	69 (17.25)	24.08	8.02	
BMI; Mean ± SD		22.46	2.98	
Underweight (<18.5)	28 (7.00)	27.21	7.73	0.198
Healthy Weight (18.5–24.9)	294 (73.50)	25.32	8.21	
Overweight and obese (≥25.00)	78 (19.50)	26.83	9.60	

BMI, Body mass index. SD, Standard deviation. BDT, Bangladeshi Taka (currency). Others, included; homemaker, unemployed, and student. *, *p* < 0.05 was considered statistically significant. The Wilcoxon rank sum test was used to measure the mean differences for variables with two categories; and the Kruskal-Wallis test was applied for variables with two or more groups. Bold values indicate statistically significant variables at the specified threshold (e.g., *p* < 0.05 or another relevant significance level).

The perceived barriers to a healthy diet score varied by gender: males scored 25.9 ± 7.65, and females scored 25.42 ± 10.07. That suggests a marginal significance (*p* = 0.052). Different family types, such as nuclear (25.87 ± 8.31), joint (24.0 ± 8.04), and life apart home (30.03 ± 9.71), revealed statistically significant differences in perceived barriers (*p* = 0.002). About 48.75% of participants had graduation degrees or higher in their educational backgrounds. Only 30.0% of the participants studied biological sciences. While 34.25% of participants held jobs, the remaining two-thirds of respondents were businesspeople and other professionals (homemakers, unemployed, and students). One-third of respondents’ working hours were 6–8; while 41.50% had no fixed working hours. Using the WHO classification, samples were divided into four BMI groups and later merged overweight and obese; which reports *n* = 28 (7.0%) being the underweight group, *n* = 294 (73.50%) normal weight group; *n* = 78 (19.50%) overweight and obese groups, respectively.

### 3.2 Mental health status of the participants

**Figure 2:**
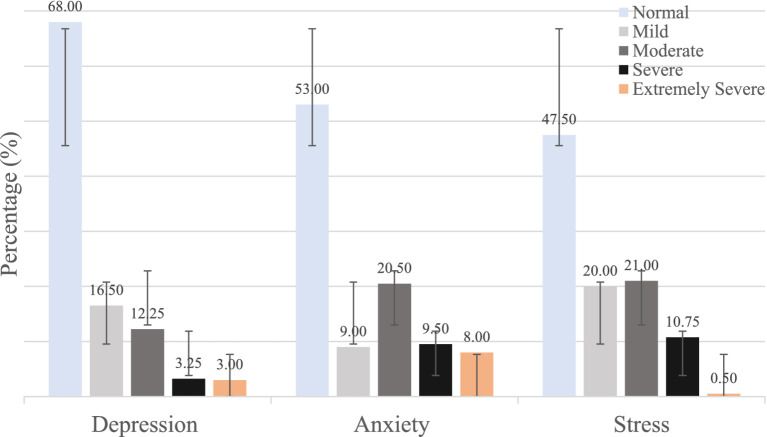
Prevalence of depression, anxiety and stress among the study participants.

Figure 2 illustrates the mental health status of the 400 participants which was obtained by the DASS-21 scale. The Mean ± SD scores for were 8.00 ± 7.22 depression, 8.62 ± 7.48 for anxiety, and 16.0 ± 7.89 for stress. The results indicated that a significant portion of the participants had mild to extremely severe levels of depression, anxiety, and stress. Notably, 32.0% of the participants reported mild to extremely severe levels of depression, 47.0% reported similar levels of anxiety, and 42.5% reported similar levels of stress.

### 3.3 Perceived barriers to healthy diet

**Figure 3:**
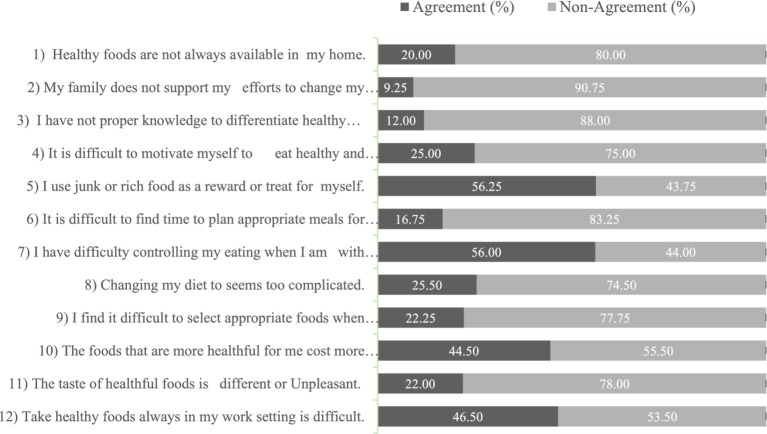
The agreements and non-agreements for perceived barriers to healthy.

Figure 3 illustrates the results regarding the percentage of agreement and non-agreement of perceived barriers to a healthy diet among the participants for each of the 12 items. The results show that 20.0% participants agreed that healthy foods were only sometimes available in their homes. Additionally, few participants (09.25%) reported that their family does not support their efforts to change their diet. A small proportion of the participants, approximately one in eight (12%), expressed the need for more knowledge about healthy foods. On the other hand, a significant number of participants (56.25%) reported consuming junk or rich food as a reward or treat. The results also showed that it is difficult for some participants to control their eating habits during an outing with friends (56.00%). More than one-quarter of the participants (25.50%) claimed that changing their diet was too complicated. Furthermore, 44.50% of individuals reported that healthful foods were more expensive than they could afford, while 22.0% of participants said that their taste was different or unpleasant. The results also showed that it took more work for some participants (46.50%) to bring healthy foods to their work setting.

### 3.4 Multivariable quantile regression analysis

Results of quantile regression, and the Ordinary Least Square Regression (OLS) models are presented in Table 2. A multivariable quantile regression was fitted on each of the 10th, 25th, 50th, 75th, and 90th quantiles of the scores for perceived barriers to healthy diet to show a complete picture of the association between the explanatory variables and perceived healthy diet barrier scores. Pseudo R^2^ values (ranging from 0.0867 to 0.2325) to reflect the model’s explanatory power across quantiles in the manuscript. These values indicate varying degrees of fit, with stronger fits observed at higher quantiles. The model estimate suggests that study location was associated with the perceived barriers to a healthy diet as people living in Chattogram were less likely to have a barrier score than those living in Dhaka city in the 25th (*β* = −3.14, 95% CI: −5.48 to −0.80) and 50th (β = −2.34, 95% CI: −4.33 to −0.34) quantile. The analysis also predicted that female gender was significantly associated with lower perceived barriers to healthy diet scores at the 50th quantile (*β* = −2.05, 95% CI: −3.88 to −0.21) after adjusting for other covariates. Respondents who lived apart from home tended to have higher scores on perceived barriers to a healthy diet compared to the nuclear family members at the 10th, 25th, and 50th quantiles. Participants who lived in a joint family were observed to be 3.17 points lower at the 10th quantile (*β* = −3.17, 95% CI: −5.45 to −0.89) perceived barriers score compared to those who lived in a nuclear family. Looking at the working hours in a day, the 90th (β = 12.44, 95% CI: 3.92 to 20.95) quantile was found to be a statistically significant predictor for the higher perceived barriers to healthy diet scores, particularly for individuals who worked for 6 h compared to the reference group.

**Table 2:** Multivariable analysis on perceived barriers to healthy diet by a quantile regression modeling along with a linear regression.

0.10 Pseudo R2 = 0.1236 0.25 Pseudo R2 = 0.0867 0.50 Pseudo R2 = 0.1308 0.75 Pseudo R2 = 0.1666 0.90 Pseudo R2 = 0.2325						
Variables	Linear regression *β* (95% CI)	10th quantile β (95% CI)	25th quantile β (95% CI)	50th quantile β (95% CI)	75th quantile β (95% CI)	90th quantile β (95% CI)
Study location						
Dhaka city (Ref)						
Chattogram city	−2.84 (−4.78 to 0.91)	−1.58 (−4.28 to 1.10)	−3.14 (−5.48 to −0.80)*	−2.34 (−4.33 to −0.34)*	−2.39 (−5.23 to 0.43)	−3.26 (−7.11 to 0.58)
Gazipur city	−0.55 (−2.60 to 1.49)	−0.41 (−3.16 to 2.33)	0.93 (−0.93 to 2.81)	0.14 (−1.19 to 1.48)	−0.64 (−3.16 to 1.87)	−1.52 (−7.42 to 4.37)
Gender						
Male (ref)						
Female	−0.91 (−2.82 to 0.98)	−1.39 (−3.70 to 0.90)	−2.25 (−4.70 to 0.19)	−2.05 (−3.88 to −0.21)*	−1.02 (−3.70 to 1.65)	2.49 (−2.24 to 7.23)
Age	0.005 (−0.09 to 0.11)	−0.02 (−0.10 to 0.05)	−0.03 (−0.11 to 0.03)	−0.006 (−0.12 to 0.11)	0.02 (−0.13 to 0.18)	0.05 (−0.17 to 0.28)
Current marital status						
Unmarried (ref)						
Married	0.72 (−1.49 to 2.94)	0.22 (−2.91 to 3.37)	−0.84 (−3.08 to 1.39)	−0.52 (−2.69 to 1.65)	0.78 (−3.16 to 4.72)	4.60 (0.37 to 8.83)
Family type						
Nuclear (ref)						
Joint	**−1.72 (−3.58 to −0.14)**	−3.17 (−5.45 to −0.89)*	−1.20 (−4.81 to 2.40)	−0.72 (−3.08 to 1.62)	−0.57 (−3.16 to 2.00)	−1.53 (−5.58 to 2.51)
Live apart home	2.43 (−0.57 to 5.43)	4.35 (0.34 to 8.35) *	3.71 (1.03 to 6.39)*	2.65 (1.25 to 4.06)*	1.25 (−1.46 to 3.97)	2.22 (−2.87 to 7.32)
Educational level						
Higher secondary and below (ref)						
Graduate and above	1.13 (−0.60 to 2.86)	1.32 (−1.09 to 3.75)	0.78 (−1.45 to 3.03)	0.10 (−1.36 to 1.56)	2.14 (0.48 to 3.80) *	3.47 (0.83 to 6.11) *
Field of study						
Biological science (ref)						
Other than biological science	−0.60 (−2.56 to 1.35)	1.68 (−0.45 to 3.83)	0.89 (−2.10 to 3.88)	−0.60 (−3.05 to 1.84)	−1.62 (−4.70 to 1.45)	−2.66 (−6.26 to 0.93)
Occupation						
Service (ref)						
Business	−2.15 (−5.00 to 0.69)	−1.01 (−4.23 to 2.19)	−0.19 (−3.60 to 3.20)	−1.69 (−4.82 to 1.43)	−1.10 (−3.86 to 1.66)	−4.33 (−9.47 to 0.79)
Others	−0.93 (−3.28 to 1.41)	1.28 (−1.56 to 4.14)	2.05 (−1.14 to 5.25)	−0.78 (−2.82 to 1.25)	−3.51 (−5.99 to −1.03)*	−3.75 (−7.75 to 0.24)
Working hours in a day						
6 Hour	5.30 (1.11 to 9.49)*	1.45 (−4.53 to 7.44)	0.38 (−7.40 to 8.16)	2.97 (−1.15 to 7.10)	5.92 (−0.21 to 12.06)	12.44 (3.92 to 20.95)*
8 Hour (ref)						
10 Hour and more	1.67 (−0.78 to 4.14)	−2.54 (−6.54 to 1.46)	−1.91 (−5.16 to 3.20)	0.79 (−1.46 to 3.06)	3.41 (−0.70 to 7.14)	3.45 (−1.95 to 8.85)
Not fixed	0.68 (−1.81 to 3.18)	−1.97(−5.29 to 1.34)	−2.52 (−6.38 to 1.33)	1.29 (−1.01 to 3.60)	1.98 (−0.43 to 4.41)	0.70 (−1.73 to 3.13)
Family monthly income (in BDT)						
<30,000	2.92 (0.53 to 5.31) *	3.87 (0.89 to 6.85)*	1.90 (−1.34 to 5.15)	1.51 (−0.20 to 3.24)	2.11 (−0.15 to 4.38)	3.01 (−0.31 to 6.35)
30,000–60,000	2.53 (0.25 to 4.81) *	3.41 (1.11 to 5.72)*	1.96 (−0.51 to 4.45)	1.89 (0.28 to 4.06)*	2.86 (0.93 to 4.79)*	2.08 (−1.29 to 5.46)
>60,000 (ref)						
BMI	−0.005 (−0.29 to 0.28)	0.14 (−0.22 to 0.50)	0.06 (−0.48 to 0.62)	−0.12 (−0.61 to 0.37)	−0.19 (−0.61 to 0.22)	−0.16 (−0.70 to 0.38)
Depression score	0.38 (0.23 to 0.52)*	−0.04 (−0.28 to 0.19)	0.10 (−0.05 to 0.26)	0.34 (0.17 to 0.51)*	0.59 (0.30 to 0.89)*	0.79 (0.49 to 1.09) *
Anxiety score	0.02 (−0.12 to 0.16)	0.05 (−0.17 to 0.27)	0.08 (−0.12 to 0.29)	0.14 (0.01 to 0.28)*	0.08 (−0.05 to 0.23)	0.09 (−0.10 to 0.29)
Stress score	0.03 (−0.09 to 0.15)	0.18 (0.09 to 0.27)*	0.12 (0.03 to 0.21)*	−0.03 (−0.12 to 0.04)	−0.07 (−0.22 to 0.07)	−0.18 (−0.45 to 0.08)

Ref, reference. CI, confidence interval. BDT, Bangladeshi Taka (currency). Symbol * was utilized to indicate statistical significance (*P*-value < 0.05). Bold values indicate statistically significant variables at the specified threshold (e.g., *p* < 0.05 or another relevant significance level).

Those whose family monthly income was (30000–60,000) BDT per month had experienced higher perceived barriers scores to healthy diet score in the 10th and 75th compared to the highest-income (> 60,000 BDT) group. The perceived barriers to a healthy diet increased by 0.34 points, 0.59 points, and 0.79 points at their 50th, 75th, and 90th quantiles, respectively, when their depression scores increased by one unit. Anxiety showed a positive and statistically significant association at the 50th quantile (*β* = 0.14, 95% CI = 0.01 to 0.28). Stress was also positively associated with perceived barrier scores at the 10th (β = 0.18, 95% CI = 0.09 to 0.27) and 25th (β = 0.12, 95% CI = 0.03 to 0.21) quantiles.

## 4 Discussion

This study aimed to explore the association of mental health status with perceived barriers to healthy diet among Bangladeshi adults. In this study, mostly reported perceived barriers to healthy diet were identified as follows: (a) using junk or rich food as a reward or treat, (b) difficulty in controlling eating when with friends, (c) The cost of healthy food being higher than what can be afforded, (d) difficulty in taking healthy food to work setting always. These findings are in line with previous studies from different counties (26, 36).

Previous literature also supports that some people may find it challenging to manage or control their eating while in social circumstances like eating with friends and relatives (37). According to a review study, the most frequent barriers to healthy eating in high income countries were unhealthy diets of friends and family members and the expectation that unhealthy food would be consumed in particular circumstances (38). Similar to our findings, evidence also revealed that sometimes people think it is too difficult to change their diet, and they think healthier items are more costly and taste different from less healthy ones (39). Prior studies have shown financial considerations are important barriers to eating healthy food but few participants claimed food prices were favorable when they were higher, or if their income was insufficient to purchase the expected amount of food (40, 41). A qualitative study consistently recognized the high price of nutritious food as a major structural barrier to eating healthy meals (42). Furthermore, the study found that it was difficult for some participants to bring healthy food always to their work setting. The CDC reported that food consumed at work is heavy in calories, salt, solid fat, added sugars, and refined carbohydrates (43). This could be a probable reason why participants perceived this as a barrier to have healthy diet.

The regression results provide insight into the relationship between various explanatory variables and perceived barriers to healthy diet. The findings indicate that study location, gender, marital status, living arrangement, working hours, family monthly income, depression, and stress are significantly associated with perceived barriers to a healthy diet in at least one of the quantiles. For instance, the model estimates suggest that being female is negatively associated with the perceived barriers to healthy diet scores at the 50th quantile. A report by Harvard Health Publishing depicted that women consume a healthier diet than males in most cases. Besides, the differences in food preferences and health awareness between males and females might explain the reason for having fewer barriers to healthy diets among females. For instance, according to a survey in Massachusetts, women were, on average, 50% more likely than males to reach the daily requirement of eating at least five servings of fruits and vegetables (44). Therefore, it appears that gender variations in perceived barriers to healthy diet could be partially explained by women’s greater engagement in weight control and partly by their stronger views of healthy eating (45).

According to the study, people in Chattogram encounter fewer barriers to consuming a healthy diet compared to those in Dhaka. Factors such as population size, urban density, and the availability of fresh food markets likely contribute to this discrepancy. Chattogram, being less crowded than Dhaka, may offer more accessible and affordable healthy food options. Research indicates that residents of smaller cities often have better access to fresh produce and fewer fast-food outlets, which could explain these differences (12).

Respondents who lived apart from home tended to have higher scores on perceived barriers to a healthy diet compared to the nuclear family members, which is consistent with the existing literature that highlights the role of family support in promoting healthy dietary behaviors (46). Another study also reported that living alone is significantly associated with a lower consumption of fruits and vegetables, where single men are more prone to eat foods that are easy to cook and prepare. This might be a plausible reason for having higher barriers to healthy diet scores among participants living apart from home (45).

Moreover, lower working hours were found to be significantly associated with higher perceived barriers to healthy diet scores. Participants who had a shorter work schedule of 6 h a day had higher scores than those who had 8-h jobs. Similarly, we also identified that participants with lower monthly income were significantly associated with higher scores of barriers to healthy diet. This indicates a connection between working hours and monthly income, where participants might have a possibility to earn less when they work limited hours. Also, our participants perceived the high cost of healthy food as a potential barrier to healthy diet. Hence it is possible that people from lower income groups may face more challenges in terms of accessibility and affordability to healthy diets (47). There is also evidence that one of the main challenges people experience when buying healthy food is the cost of food (48). A mixed-method study argued that when participants were asked about their work schedules and commute hours, several participants reported that they found it challenging to make healthy diet because of their schedules and working hours (49).

Psychological distress like depressive, anxiety and stress symptoms were found to be significantly associated with perceived barriers to healthy diet scores, which supports previous research highlighting the negative impact of mental health on health behaviors (50). Another study shows that people with mental illness who experienced barriers to healthy eating and exercise have a difficult timing to living a healthy lifestyle (35). There is a strong link between diet and mental health status (51), where it is evident that adherence to dietary recommendations results in a sufficient intake of nutrients and can lower risk and lessen the symptoms of mental illness (52). People with depression are more prone to consume more calories and eat unhealthy foods (53). Evidence also suggests that people who have higher degrees of psychological distress are less careful in choosing their food and tend to eat more and in larger portions than they need to, thus controlling their emotions via food (54). Collectively these could be the triggering reasons why individuals with symptoms of mental health issues were more likely to perceive higher scores regarding the barriers to healthy diet.

### 4.1 Policy implications

To address the identified barriers to a healthy diet among adults, policymakers, healthcare professionals, public health organizations, and stakeholders should take note of the study’s findings and initiate targeted interventions. Based on the findings, suggesting targeted interventions that address both mental health and dietary barriers could be a practical application for improving public health in Bangladesh. Advocacy for policies supporting mental health services and stress management programs is crucial, as they significantly facilitate healthier food choices. Education campaigns aimed at raising awareness about the importance of a balanced diet and its connection to mental health could play a pivotal role. These campaigns should be designed to reach diverse populations, including underserved communities, to ensure equitable access to information. Workplace interventions, such as the incorporation of healthy meal programs, stress management workshops, and access to mental health resources, are also recommended. Further research on innovative approaches like healthy food labeling systems and food technology-based interventions is necessary to enhance the effectiveness of interventions addressing barriers to a healthy diet. Future recommendations for research could include examining the effectiveness of these targeted interventions in addressing the perceived barriers to healthy diet and identifying additional factors that may influence healthy eating behavior among adults. Incorporating qualitative methods, such as interviews or focus groups, can offer deeper insights into cultural and contextual factors shaping dietary behavior, complementing quantitative findings to refine interventions.

### 4.2 Strengths and limitations

This study evaluated participants’ mental health status and its association with perceptions of barriers to adopting a healthy diet. It also explores a specific context, providing insights into barriers in a representative study setting. Furthermore, this study looks into the relationship between these barriers and mental health, contributing a unique perspective on the interplay between diet and mental health. These findings contribute to the growing understanding of how barriers to healthy diets can impact overall well-being. Since the outcome variable was not linearized and not normally distributed, we used a robust statistical technique, quantile regression, to determine the association between barriers to healthy diet and other covariates. Nonetheless, this study has a few limitations. Given that it was a cross-sectional study; it was not possible to determine if certain factors caused the reported barriers to a healthy diet. The reliance on self-reported data to assess dietary habits introduces the risk of recall bias, as participants may have difficulty accurately recalling their food intake or may alter their responses. This could affect the reliability of the dietary data. Additionally, potential participant error is another concern, as misunderstandings or socially desirable responses may influence the accuracy of the reported information. Furthermore, the study’s sample was drawn from three large cities in Bangladesh, which limits its generalizability, especially to rural areas where dietary habits and health behaviors may differ significantly. Finally, the use of a non-validated questionnaire to assess dietary barriers is another limitation, as it may not accurately capture the relevant factors affecting participants’ diets.

## 5 Conclusion

This study highlights a significant association between mental health status and perceived barriers to maintaining a healthy diet among adults in Bangladesh. The identified barriers include using junk food as a reward, the inability to control eating in social situations, the high cost of healthy food, the difficulty of bringing healthy food to the workplace, and motivational issues. The findings underscore the need for specific strategies to overcome these barriers, such as promoting healthier food choices in social settings, increasing the affordability and accessibility of nutritious foods, and integrating mental health support.

Future research should focus on culturally tailored nutritional counseling, workplace-based healthy eating programs, and mental health-focused dietary interventions. Longitudinal studies could clarify the causal relationship between mental health and dietary behavior, while qualitative research could explore personal experiences related to dietary challenges.

## Data Availability

The raw data supporting the conclusions of this article will be made available by the authors, without undue reservation.

## References

[1] Fanzo J Drewnowski A Blumberg J Miller G Kraemer K. Nutrients, foods, diets, people: Promoting healthy eating. (2020) 1–11. 10.1093/cdn/nzaa069 PMC7250582 32494761

[2] Cena H Calder PC. Defining a healthy diet: evidence for the role of contemporary dietary patterns in health and disease. Nutrients. (2020) 12:1–15. doi: 10.3390/nu12020334, PMID: 32012681 PMC7071223

[3] Brushett S de Kroon MLA Katsas K Engel O Reijneveld SA Linos A. Healthy diets positively associated with health-related quality of life in children and adolescents from low socioeconomic areas: Findings from the Greek Food Aid Program, DIATROFI. Nutrition. (2024) 121:112367:112367. doi: 10.1016/j.nut.2024.112367 38428360

[4] Li D Jia Y Yu J Liu Y Li F Liu Y. Adherence to a healthy lifestyle and the risk of all-cause mortality and cardiovascular events in individuals with diabetes: the ARIC study. Front Nutr. (2021) 8:1–10. doi: 10.3389/fnut.2021.698608, PMID: 34291073 PMC8287067

[5] The State of Food Security and Nutrition in the World. (2022). The State of Food Security and Nutrition in the World 2022.

[6] Dizon F Wang Z Mulmi P. The cost of a nutritious diet in Bangladesh, Bhutan, India, and Nepal. Policy res work pap—World Bank. (2021). Available at: http://www.worldbank.org/prwp

[7] Hallum SH Hughey SM Wende ME Stowe EW Kaczynski AT. Healthy and unhealthy food environments are linked with neighbourhood socio-economic disadvantage: an innovative geospatial approach to understanding food access inequities. Public Health Nutr. (2020) 23:3190. doi: 10.1017/S1368980020002104 32782060 PMC10200448

[8] Rakhshanda S Barua L Faruque M Banik PC Shawon RA Rahman AKMF. Malnutrition in all its forms and associated factors affecting the nutritional status of adult rural population in Bangladesh: results from a cross-sectional survey. BMJ Open. (2021) 11:e051701. doi: 10.1136/bmjopen-2021-051701, PMID: 34706956 PMC8552130

[9] Wongprawmas R Sogari G Menozzi D. Strategies to promote healthy eating among university students: a qualitative study using the nominal group technique. Front Nutr. (2022) 9:9. doi: 10.3389/fnut.2022.821016 PMC8847783 35187039

[10] Deslippe AL Soanes A Bouchaud CC Beckenstein H Slim M Plourde H. Barriers and facilitators to diet, physical activity and lifestyle behavior intervention adherence: a qualitative systematic review of the literature. Int J Behav Nutr Phys Act. (2023) 20:14–25. doi: 10.1186/s12966-023-01424-2, PMID: 36782207 PMC9925368

[11] Yarmohammadi S Saadati HM Ghaffari M Ramezankhani A. A systematic review of barriers and motivators to physical activity in elderly adults in Iran and worldwide. Epidemiol Health. (2019) 41:1–11. doi: 10.4178/epih.e2019049, PMID: 31801319 PMC6976727

[12] Hasan AMR Smith G Rashid H Selim MA Rasheed S. Promoting healthy foods among urban school children in Bangladesh: a qualitative inquiry of the challenges and opportunities. BMC Public Health. (2021) 21:1–12. doi: 10.1186/s12889-021-11085-0, PMID: 34074273 PMC8168019

[13] Bangladesh IPC Chronic Food Insecurity Report (2022)—Bangladesh | ReliefWeb. [cited 2022 Sep 2]. Available at: https://reliefweb.int/report/bangladesh/bangladesh-ipc-chronic-food-insecurity-report-june-2022

[14] Zingel W-P Keck M Etzold B Bohle H-G. Urban food security and health status of the poor in Dhaka, Bangladesh In: Krämer A Khan MH Kraas F, editors. Health in megacities and urban areas. Heidelberg: Physica-Verlag HD (2011). 301–19.

[15] Hossain MD Ahmed HU Chowdhury WA Niessen LW Alam DS. Mental disorders in Bangladesh: a systematic review. BMC Psychiatry. (2014) 14:1–8. doi: 10.1186/s12888-014-0216-9, PMID: 25073970 PMC4149198

[16] World Health Organization. Bangladesh WHO special initiative for mental health situational assessment. Geneva, Switzerland: World Health Organization (2021).

[17] Lu Q Wang Y Geng T Zhang Y Tu Z Pan A. Depressive symptoms, lifestyle behaviors, and risk of cardiovascular disease and mortality in individuals of different socioeconomic status: a prospective cohort study. J Affect Disord. (2024) 347:345–51. doi: 10.1016/j.jad.2023.11.046, PMID: 37989438

[18] Solomou S Logue J Reilly S Perez-Algorta G. A systematic review of the association of diet quality with the mental health of university students: implications in health education practice. Health Educ Res. (2023) 38:28–68. doi: 10.1093/her/cyac035, PMID: 36441584 PMC9853940

[19] Eliby D Simpson CA Lawrence AS Schwartz OS Haslam N Simmons JG. Associations between diet quality and anxiety and depressive disorders: A systematic review. J Affect Dis Rep. (2023) 14:100629:100629. doi: 10.1016/j.jadr.2023.100629, PMID: 39915132

[20] Keck MM Vivier H Cassisi JE Dvorak RD Dunn ME Neer SM. Examining the role of anxiety and depression in dietary choices among college students. Nutrients. (2020) 12:1–19. doi: 10.3390/nu12072061 PMC7400947 32664465

[21] Collins S Dash S Allender S Jacka F Hoare E. Diet and mental health during emerging adulthood: a systematic review. Emerg Adulthood. (2022) 10:645–59. doi: 10.1177/2167696820943028

[22] Selvaraj R Selvamani Y Zahra A Malla J Dhanoa RK Venugopal S. Association between dietary habits and depression: a systematic review. Cureus. (2022) 14:e32359. doi: 10.7759/cureus.32359 36632273 PMC9828042

[23] Calcaterra V Rossi V Magenes VC Baldassarre P Grazi R Loiodice M. Dietary habits, depression and obesity: an intricate relationship to explore in pediatric preventive strategies. Front Pediatr. (2024) 12:1368283. doi: 10.3389/fped.2024.1368283, PMID: 38523835 PMC10957686

[24] Subhani S Grace C Begum R Kopelman P Greenhalgh T. Understanding barriers to healthy lifestyles in a Bangladeshi community. J Diabetes Nurs. (2009) 13:58–66.

[25] Andajani-Sutjahjo S Ball K Warren N Inglis V Crawford D. Perceived personal, social and environmental barriers to weight maintenance among young women: a community survey. Int J Behav Nutr Phys Act. (2004). 1–15. doi: 10.1186/1479-5868-1-15, PMID: 15462679 PMC524367

[26] Musaiger AO Al-Mannai M Tayyem R Al-Lalla O Ali EYA Kalam F. Perceived barriers to healthy eating and physical activity among adolescents in seven Arab countries: a cross-cultural study. Sci World J. (2013) 2013:232164. doi: 10.1155/2013/232164, PMID: 24348144 PMC3848306

[27] El-Bagoury LS Hassan AM AbouSeif HA. Eating attitudes and barriers to healthy eating and physical activity among a sample of university students in Egypt. J Egypt Public Health Assoc. (2017) 92:29–35. doi: 10.21608/EPX.2018.6650, PMID: 29924925

[28] Osman A Wong JL Bagge CL Freedenthal S Gutierrez PM Lozano G. The depression anxiety stress Scales-21 (DASS-21): further examination of dimensions, scale reliability, and correlates. J Clin Psychol. (2012) 68:1322–38. doi: 10.1002/jclp.21908, PMID: 22930477

[29] Alim SAHM Kibria SME Lslam MJ Uddin MZ Nessa M Wahab MA. Translation of DASS 21 into Bangla and validation among medical students. Bangladesh J Psychiatry. (2017) 28:67–70. doi: 10.3329/bjpsy.v28i2.32740

[30] Henry JD Crawford JR. The short-form version of the depression anxiety stress scales (DASS-21): construct validity and normative data in a large non-clinical sample. Br J Clin Psychol. (2005) 44:227–39. doi: 10.1348/014466505X29657 16004657

[31] Lovibond PF Lovibond SH. The structure of negative emotional states: comparison of the depression anxiety stress scales (DASS) with the Beck depression and anxiety inventories. Behav Res Ther. (1995) 33:335–43. doi: 10.1016/0005-7967(94)00075-U 7726811

[32] Body mass index (BMI). [cited 2023 Mar 1]. (2023). Available at: https://www.who.int/data/gho/data/themes/topics/topic-details/GHO/body-mass-index

[33] Yu K Moyeed RA. Bayesian quantile regression. Stat Probab Lett. (2001) 54:437–47. doi: 10.1016/S0167-7152(01)00124-9

[34] Koenker R. Quantile regression. Quantile Regres (2005) [cited 2024 Aug 26]; 1–349. Available at: https://www.cambridge.org/core/books/quantile-regression/C18AE7BCF3EC43C16937390D44A328B1

[35] Hasan ABMN Bin SA Jahan I. Perceived barriers to maintain physical activity and its association to mental health status of Bangladeshi adults: a quantile regression approach. Sci Rep. (2023) 13:8993–13. doi: 10.1038/s41598-023-36299-7, PMID: 37268704 PMC10238517

[36] Pico ML Grunnet LG Vinter CA Aagaard-Hansen J Kragelund Nielsen K. Barriers and facilitators for sustainable weight loss in the pre-conception period among Danish women with overweight or obesity – a qualitative study. BMC Public Health. (2023) 23:1778. doi: 10.1186/s12889-023-16676-7, PMID: 37704956 PMC10500859

[37] Liu KSN Chen JY Ng MYC Yeung MHY Bedford LE Lam CLK. How does the family influence adolescent eating habits in terms of knowledge, attitudes and practices? A global systematic review of qualitative studies. Nutrients. (2021) 13:3717. doi: 10.3390/nu13113717, PMID: 34835973 PMC8624651

[38] Munt AE Partridge SR Allman-Farinelli M. The barriers and enablers of healthy eating among young adults: a missing piece of the obesity puzzle: a scoping review. Obes Rev. (2017) 18:1–17. doi: 10.1111/obr.12472, PMID: 27764897

[39] Domosławska-Żylińska K Łopatek M Krysińska-Pisarek M Sugay L. Barriers to adherence to healthy diet and recommended physical activity perceived by the polish population. J Clin Med. (2024) 13:22. doi: 10.3390/jcm13010022, PMID: 38202029 PMC10779332

[40] Amstutz D Gonçalves D Hudelson P Stringhini S Durieux-Paillard S Rolet S. Nutritional status and obstacles to healthy eating among refugees in Geneva. J Immigrant Minority Health. (2020) 22:1126–34. doi: 10.1007/s10903-020-01085-4, PMID: 32940816 PMC7683482

[41] Van Der Velde LA Schuilenburg LA Thrivikraman JK Numans ME Kiefte-De Jong JC. Needs and perceptions regarding healthy eating among people at risk of food insecurity: a qualitative analysis. Int J Equity Health. (2019) 18:1–12. doi: 10.1186/s12939-019-1077-0, PMID: 31775770 PMC6880580

[42] Tiedje K Wieland ML Meiers SJ Mohamed AA Formea CM Ridgeway JL. A focus group study of healthy eating knowledge, practices, and barriers among adult and adolescent immigrants and refugees in the United States. Int J Behav Nutr Phys Act. (2014) 11:1–16. doi: 10.1186/1479-5868-11-63, PMID: 24886062 PMC4030459

[43] CDC. Healthy food environments: Improving access to healthier food. Centers for Disease Control and Prevention (CDC) (2020).

[44] Harvard Health Publishing. Mars vs. Venus In: The gender gap in health. Harvard Medical School (2019)

[45] Feraco A Armani A Amoah I Guseva E Camajani E Gorini S. Assessing gender differences in food preferences and physical activity: a population-based survey. Front Nutr. (2024) 11:1348456. doi: 10.3389/fnut.2024.1348456, PMID: 38445208 PMC10912473

[46] Schnettler B Lobos G Miranda-Zapata E Denegri M Ares G Hueche C. Diet quality and satisfaction with life, family life, and food-related life across families: a cross-sectional pilot study with mother-father-adolescent triads. Int J Environ Res Public Health. (2017) 14:1–24. doi: 10.3390/ijerph14111313, PMID: 29109387 PMC5707952

[47] Wolfson JA Ramsing R Richardson CR Palmer A. Barriers to healthy food access: associations with household income and cooking behavior. Prev Med Rep. (2019) 13:298–305. doi: 10.1016/j.pmedr.2019.01.023, PMID: 30792944 PMC6363918

[48] Ver Ploeg M Dutko P Breneman V. Measuring food access and food deserts for policy purposes. Appl Econ Perspect Policy. (2015) 37:205–25. doi: 10.1093/aepp/ppu035

[49] Emma DF Rachel L Louisa P Bethan J Melanie G Angela W. Perceived barriers and facilitators of exercise and healthy dietary choices: A study of employees and managers within a large transport organisation. SAGE Publications. (2017) 1:1–29.

[50] Lachance L Ramsey D. Food, mood, and brain health: implications for the modern clinician. Mo Med. (2015) 112:111–5. PMID: 25958655 PMC6170050

[51] Kundu S Rejwana N Al Banna MH Kawuki J Ghosh S Alshahrani NZ. Linking depressive and anxiety symptoms with diet quality of university students: A cross-sectional study during the COVID-19 pandemic in India. Healthcare. (2022) 10:1848. doi: 10.3390/healthcare10101848 36292298 PMC9602108

[52] Ljungberg T Bondza E Lethin C. Evidence of the importance of dietary habits regarding depressive symptoms and depression. Int J Environ Res Public Health. (2020) 17. doi: 10.3390/ijerph17051616 PMC7084175 32131552

[53] Kenney E Rampalli KK Samin S Frongillo EA Reyes LI Bhandari S. How livelihood change affects food choice behaviors in low- and middle-income countries: a scoping review. Adv Nutr. (2024) 15:100203. doi: 10.1016/j.advnut.2024.100203, PMID: 38462217 PMC11007434

[54] Braden A Musher-Eizenman D Watford T Emley E. Eating when depressed, anxious, bored, or happy: are emotional eating types associated with unique psychological and physical health correlates? Appetite. (2018) 125:410–7. doi: 10.1016/j.appet.2018.02.022, PMID: 29476800

