# Supplementary material for: Association of mental health status with perceived barriers to healthy diet among Bangladeshi adults: a quantile regression-based approach
Source: Front Public Health. 2025 Feb 19;13:1487107. doi: 10.3389/fpubh.2025.1487107 (PMC11879812; doi:10.3389/fpubh.2025.1487107)
Supplement: Supplementary file 1 [file Data_Sheet_1.pdf]

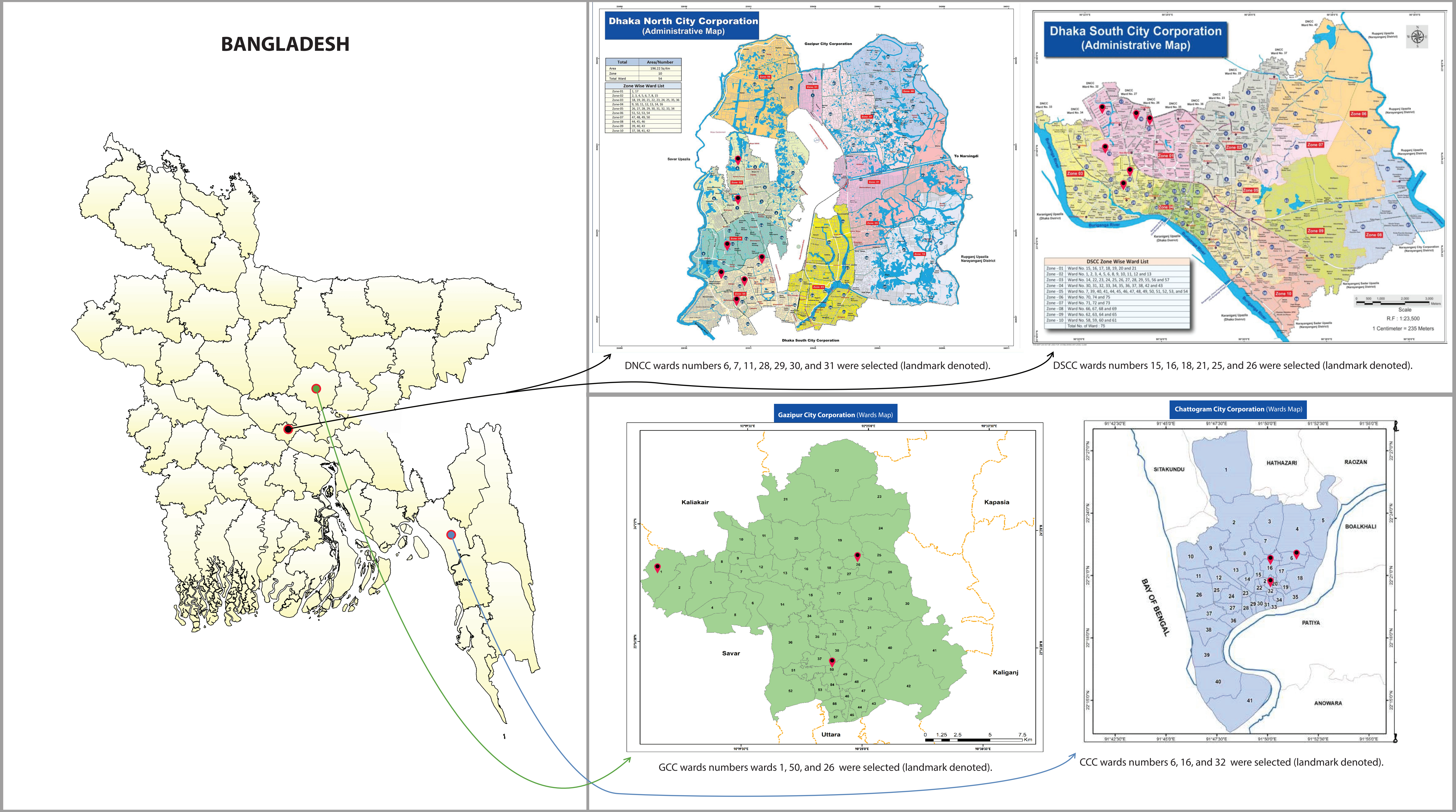
BANGLADESH
Dhaka North City Corporation
(Administrative Map)
Gazipur City Corporation
Savar Upazila
To Narsingdi
Dhaka South City Corporation
DNCC wards numbers 6, 7, 11, 28, 29, 30, and 31 were selected (landmark denoted).
Dhaka South City Corporation
(Administrative Map)
Buriganga River
DSCC Zone Wise Ward List
Scale
R.F : 1:23,500
1 Centimeter = 235 Meters
DSCC wards numbers 15, 16, 18, 21, 25, and 26 were selected (landmark denoted).
Gazipur City Corporation (Wards Map)
Kaliakair
Kapasia
Savar
Kaliganj
Uttara
GCC wards numbers wards 1, 50, and 26 were selected (landmark denoted).
Chattogram City Corporation (Wards Map)
SITAKUNDU
HATHAZARI
RAOZAN
BOALKHALI
BAY OF BENGAL
PATIYA
ANOWARA
CCC wards numbers 6, 16, and 32 were selected (landmark denoted).
